# Guardians of the blood: How Kupffer cells control systemic infection

**DOI:** 10.1371/journal.ppat.1014744

**Published:** 2026-10-30

**Authors:** Lindsey Orthner, Josefien W. Hommes, Bas G. J. Surewaard

**Affiliations:** Department of Microbiology, Immunology, and Infectious Disease, Snyder Institute for Chronic Diseases, University of Calgary, Calgary, Alberta, Canada; Duke University School of Medicine, UNITED STATES OF AMERICA

## Introduction

Bloodstream infections represent a critical failure of host barrier defenses and can rapidly progress to life-threatening sepsis. Once microbes enter the circulation, the host must detect and remove them before they seed distant tissues. The liver is ideally positioned for this task: blood from both the portal and systemic circulation passes through a dense network of slow-flowing sinusoids lined by Kupffer cells (KCs), specialized liver-resident macrophages that continuously survey the vascular compartment. Rather than acting simply as tissue macrophages, KCs function as intravascular sentinels that capture microbes under flow, kill internalized organisms, and recruit additional immune cells when containment is threatened, largely as established in murine infection models given the constraints of studying bloodstream pathogen capture directly in humans. Yet this defense is not absolute. Several pathogens avoid capture or survive within KCs, converting a protective niche into a potential reservoir for dissemination. Here, we highlight five concepts that define how KCs protect the bloodstream and how pathogens circumvent this hepatic immune firewall (Fig 1).

**Fig 1:**
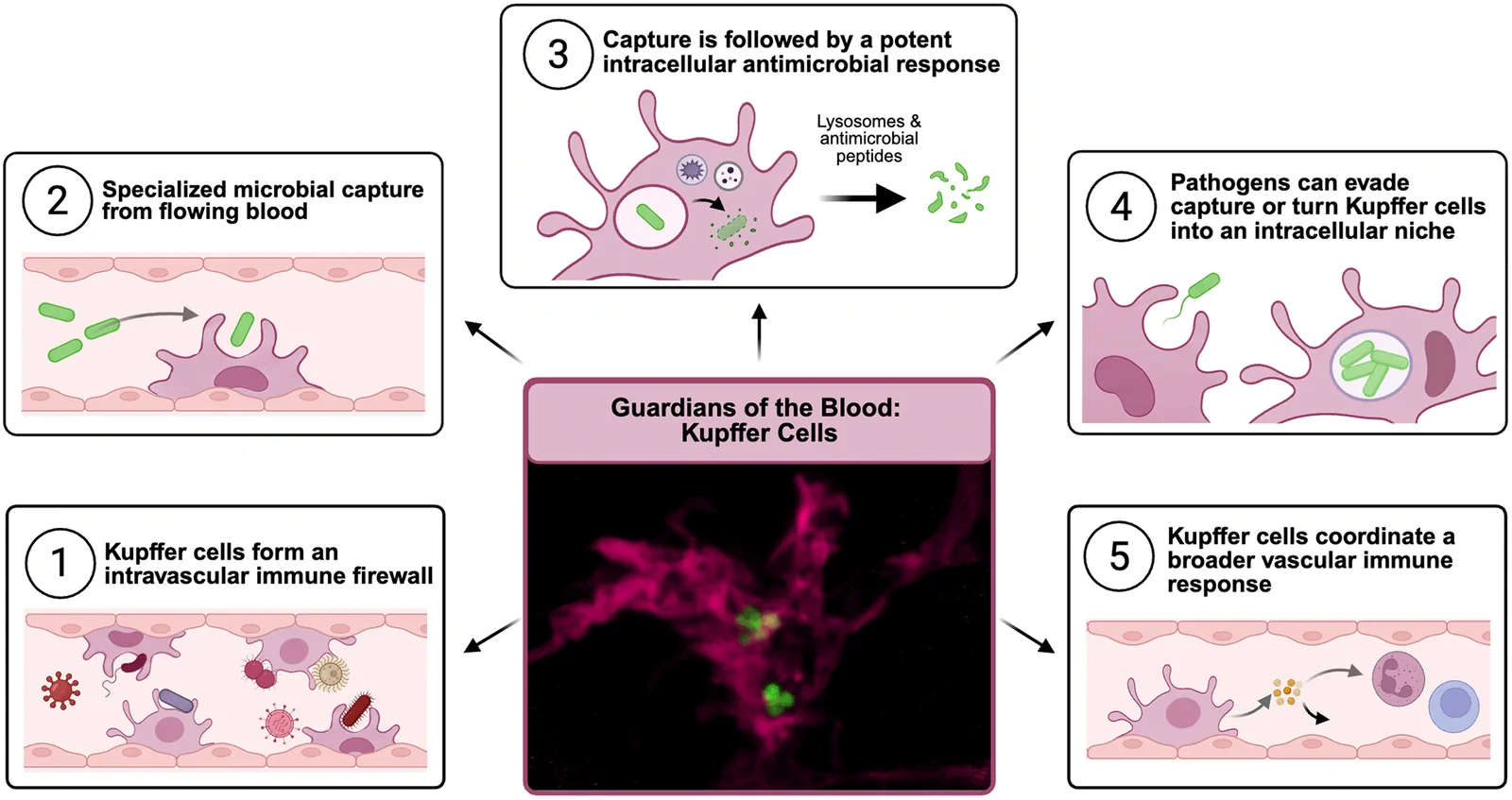
Five concepts defining Kupffer cell-mediated protection of the bloodstream. Central micrograph: a 100X confocal Z-stack of a single Kupffer cell (magenta) that has captured bacteria (green) in the hepatic sinusoid, demonstrating the bacterial capture that serves as the basis for the five concepts shown. (1) Kupffer cells are located along the sinusoidal endothelium (light red), where they catch various types of pathogens (purple, red, and beige) in circulation before they disseminate to other organs. (2) Kupffer cells have specialized receptors that allow capture of bacteria despite the shear forces of flowing blood. (3) Captured bacteria are trafficked into phagolysosomes that contain antimicrobial peptides, hydrolytic enzymes, and reactive oxygen and nitrogen species that create a hostile environment for bacterial killing. (4) Certain pathogens can evade Kupffer cell capture, whereas others can survive within, using them as an intracellular niche to survive and replicate. (5) Activated Kupffer cells amplify the vascular immune response by recruiting additional immune cells such as neutrophils and lymphocytes. Image was created using BioRender: https://BioRender.com/6y3n9nf.

## Concept 1: Kupffer cells form an intravascular immune firewall

KCs are the largest population of tissue-resident macrophages and are strategically positioned along the sinusoidal endothelium, where they are directly exposed to circulating blood. Their sessile location and ability to extend cellular processes into the sinusoidal lumen make them particularly well-suited to intercept microbes moving through the vasculature [1]. This anatomical arrangement is functionally important: KC depletion markedly reduces hepatic trapping and allows pathogens to remain in circulation and disseminate to distant organs. This phenotype has been observed across a wide range of pathogens, including Gram-positive and Gram-negative bacteria such as *Staphylococcus aureus*, *Escherichia coli*, and *Klebsiella pneumoniae*, as well as fungal and parasitic infections [2–4].

Although KCs originate predominantly from embryonic progenitors and self-renew during homeostasis, the hepatic niche continuously instructs their phenotype. Signals from liver sinusoidal endothelial cells, stellate cells, hepatocytes, and the gut environment shape KC identity and antimicrobial function [5,6]. During niche disruption, including infection or injury, circulating monocytes can enter the liver and acquire KC-like features in response to the local niche, TGF-β/BMP, and metabolic signals [5].

The intravascular positioning of KCs itself is dynamic. During early postnatal development, KCs acquire their mature luminal localization, increasing their capacity to capture circulating bacteria such as *E. coli* [7]. During liver injury and fibrosis, KCs can also adapt morphologically, including the formation of enlarged or multinucleated cells that may enhance bacterial trapping in altered vascular environments [8]. Together, these observations support a view of KCs as a specialized and adaptable immune firewall positioned at a major vascular checkpoint.

## Concept 2: Specialized microbial capture from flowing blood

Pathogen capture in the bloodstream presents a different challenge from uptake under static conditions; microbes must first be arrested despite vascular shear forces. The architecture of hepatic sinusoids facilitates this process through slow, discontinuous blood flow that increases contact between circulating pathogens and KCs. KC distribution within the sinusoidal network further favors early interception of microbes entering the liver [9].

Capture mechanisms are strongly pathogen-dependent. KCs express complement, scavenger, and Fcγ receptors, but one particularly important receptor is the Complement Receptor of the Immunoglobulin superfamily (CRIg; VSIG4). CRIg mediates bacterial binding and uptake under both opsonized and non-opsonized conditions, functioning through complement component C3 and direct recognition of lipoteichoic acid on Gram-positive bacteria, respectively [10,11]. It is uniquely capable of supporting capture under high-flow dynamics of the sinusoidal circulation, a property not shared by other complement receptors. Gram-negative bacterial capture is more dependent on C3 opsonization, although the relevant complement receptor(s) remain incompletely defined. Natural antibodies, C-reactive protein, and lectin pathways such as the Ashwell-Morell receptor further contribute to pathogen recognition in a microbe- and context-dependent manner [12–14].

Adaptive immunity can reinforce this innate filtration system. Vaccine-induced antibodies directed against bacterial surface structures promote opsonization and complement deposition, thereby increasing KC-mediated arrest. This may be particularly important for encapsulated organisms such as *Streptococcus pneumoniae* or *Salmonella* spp., consistent with vaccine efficacy in humans, for which surface structures can otherwise limit recognition [15,16]. Thus, the efficiency of hepatic filtration reflects both the physical properties of the sinusoidal circulation and a pathogen-specific repertoire of innate and antibody-dependent recognition mechanisms.

## Concept 3: Capture is followed by a potent intracellular antimicrobial response

Once a bacterium is captured, containment is only the first step. Internalized microbes are trafficked into the phagosome that rapidly matures through fusion with lysosomes, generating acidic, enzyme-rich compartments capable of degrading microbial material called phagolysosomes. Hydrolytic enzymes, phagosomal acidification, reactive oxygen species (ROS) and reactive nitrogen species together create a hostile intracellular environment to kill microbes [17].

Among these antimicrobial mechanisms, NADPH oxidase–dependent ROS production is particularly important for controlling *S. aureus*. Impairment of this pathway results in markedly increased intracellular bacterial replication within KCs, whereas other antimicrobial pathways appear to play more limited roles, illustrating that efficient vascular capture alone is insufficient when intracellular killing is compromised [2]. Pattern recognition and inflammatory signaling can further enhance antimicrobial activity by promoting phagosome maturation and cytokine production.

Under homeostatic conditions, the liver is not a constitutively bacteria-containing organ despite its exposure to portal blood. This emphasizes the efficiency with which the hepatic filtration system captures and destroys microbes that reach the circulation. Nevertheless, intracellular killing is not uniformly sterilizing. The outcome depends on both the antimicrobial state of the KC and the ability of the invading organism to withstand or manipulate phagolysosomal formation or environment.

## Concept 4: Pathogens can evade capture or turn Kupffer cells into an intracellular niche

The effectiveness of the KC firewall varies significantly among pathogens. Some organisms are efficiently captured and destroyed, whereas others avoid initial recognition or survive after phagocytosis [7,16,18]. These strategies can be considered two distinct routes around the same defense: remaining invisible to the KC or surviving once inside.

Bacterial surface architecture is a major determinant of capture. Capsules can reduce recognition and phagocytosis; for example, encapsulated *S. pneumoniae and K*. *pneumoniae* can evade hepatic capture more effectively than capsule-deficient bacteria [12,19]. Other bacterial surface structures can potentially alter the probability that a circulating bacterium will be arrested in the liver. These examples highlight that the bloodstream phase of infection is not passive transit: pathogen surface composition directly determines interactions with the hepatic vascular immune system.

Other pathogens are readily captured but have evolved distinct strategies to resist intracellular killing. *S. aureus* provides a striking example. Although most circulating bacteria are rapidly sequestered and killed by KCs, a fraction survives and replicates within acidified, LAMP-1-positive phagolysosomes, establishing an intracellular reservoir [18]. Subsequent KC death releases viable bacteria back into the circulation, promoting dissemination to secondary organs such as the kidneys. In contrast*, Listeria monocytogenes* escapes KC capture by using listeriolysin O to perforate the KC membrane, allowing it to re-enter the hepatic sinusoids and reach hepatocytes and replicate [20]. There, Listeria exploits ActA-dependent host actin polymerization to drive intracellular motility and direct cell-to-cell spread, disseminating between neighboring hepatocytes while minimizing extracellular exposure [21]. Thus, pathogens can undermine the KC firewall through fundamentally different intracellular strategies.

## Concept 5: Kupffer cells coordinate a broader vascular immune response

KCs do more than directly ingest microbes. Once activated, they serve as signaling hubs that recruit and organize additional immune cells within the hepatic vasculature. KC-derived chemokines, including CXCL1 and CXCL2, promote rapid neutrophil recruitment to liver sinusoids, bringing highly antimicrobial effector cells into close proximity to captured pathogens [22,23].

This cooperation is spatially organized. KCs upregulate ICAM-1 and promote integrin activation on neutrophils, enabling adhesion that allows neutrophils to accumulate around infected KCs and provide additional antimicrobial capacity when resident macrophages alone are insufficient [22]. Platelets can also accumulate at sites of infection and interact with KCs, contributing to vascular containment and promoting neutrophil extracellular trap (NET) formation [24]. Together, these multicellular interactions transform the hepatic sinusoid from a simple vascular filter into a dynamic immune compartment.

KCs can also coordinate lymphocyte responses. As antigen-presenting cells, KCs can process and present bacterial antigens, though this capacity is limited under homeostatic conditions, likely contributing to hepatic tolerance toward gut-derived antigens [25]. During infection, however, KCs become particularly effective at activating invariant killer T (iNKT) cells through CD1d-restricted antigen presentation: during *Borrelia burgdorferi* infection, for example, KCs capture circulating spirochetes and recruit iNKT cells, which cluster around infected KCs and become activated in this CD1d-dependent manner [26].

However, this amplified immune response requires careful regulation. While recruitment of additional effector cells enhances microbial containment, excessive vascular inflammation can contribute to tissue injury. Understanding how KCs balance these protective and pathological responses will be important for defining their role during systemic infection.

## Conclusion

Kupffer cells occupy a unique position at the interface between the circulation and tissue immunity. Their ability to arrest microbes under flow, kill internalized organisms, and rapidly recruit additional immune effectors makes the liver a powerful barrier against systemic dissemination. At the same time, pathogen-specific mechanisms that prevent capture or permit intracellular survival reveal important vulnerabilities in this defense system, largely defined through the murine models discussed above. Defining how microbial surface structures, antibodies, the hepatic microenvironment, and KC antimicrobial programs determine these outcomes should provide a more complete understanding of bloodstream infection and may reveal opportunities to strengthen host defense without amplifying damaging inflammation. Future work combining intravital imaging with human liver tissue or organoid models will be important for translating these mechanistic insights into therapies that enhance KC-mediated capture or limit intracellular bacterial survival.
